# Matters Arising: Safety of SARS-CoV-2 vaccination during pregnancy - obstetric outcomes from a large cohort study: methodological biases in study design with potential impact on the study’s interpretation

**DOI:** 10.1186/s12884-025-07784-w

**Published:** 2025-07-01

**Authors:** Retsef Levi, Efrat Schurr

**Affiliations:** 1 Massachusetts Institute of Technology, Cambridge, United States; 2 Pediatrician, Jerusalem, Israel

In the paper “Safety of SARS-CoV-2 vaccination during pregnancy - obstetric outcomes from a large cohort study”, BMC Pregnancy and Childbirth 22 [1], Dick et al. compare the retrospective maternal and neonatal outcomes of vaccinated and unvaccinated women, who delivered in a large tertiary care center in Jerusalem, Israel, during the period December 2020 throughout July 2021. Two of the main outcomes in their study are ‘Preterm birth’ (prior to gestational week 37) and ‘intrauterine fetal demise’ (stillbirth) rates.

The authors find no statistical difference between the respective rates of these two outcomes among vaccinated and unvaccinated women with singleton pregnancy and no history of prior SARS-CoV-2 infection (0.87% and 1% stillbirth rate and 5.5% and 6.2% preterm birth rate for vaccinated and unvaccinated women, respectively). They optimistically conclude that “SARS-CoV-2 appears to be safe during pregnancy” since there is no association between vaccination during pregnancy and negative maternal and neonatal outcomes. They do acknowledge however, that women who first vaccinated in the second trimester had a higher rate of preterm birth compared to unvaccinated women, and hypothesized that there could be “unmeasured confounding that may contribute to the result”. They also acknowledge the retrospective design as a limitation of the study and call for further studies to address some of the limitations.

The purpose of this Matters Arising is to highlight that the analysis by Dick et al. seems to have several inherent biases with potentially significant impact on the interpretation of the results.

## Controlling for gestational age

The first bias in the study is that the analysis does not control for gestational age. This is particularly important since the risk of both stillbirths and preterm deliveries is decreasing with gestational age. For example, statistics published by the Israel Ministry of Health (IMOH) [2] indicate that 51% of stillbirths occur before week 28, 15% occur between weeks 28–32, 17% occur between weeks 33–36, 12% occur between weeks 37–39 and only 5% occur post week 39.

Indeed, a priori, both vaccinated and unvaccinated women experience decreased risk as they advance in their pregnancy. However, the source of the bias stems from the fact that the exposure (i.e., vaccination) is confounded with a reduced risk of a negative outcome through the gestational age. Specifically, as the data in Dick et al. suggest, most women in Israel were first vaccinated at later stages of pregnancy with 12, 964 and 1329 women who received the first dose during the first, second and third trimester, respectively. Moreover, the study includes only women who received two doses three weeks apart^1^. Thus, the mere fact that a woman was vaccinated increases the likelihood that she arrived to a more advanced gestational age, and therefore had a reduced risk of preterm birth or stillbirth. This creates a fundamental bias since vaccinated women, who are likely to be vaccinated at advanced gestational age, and therefore have, at that point in time, a reduced risk, are compared to unvaccinated women at all gestational ages.

This bias is also illustrated by the data provided by Dick et al. that show that the stillbirth rate among women who received the first dose in the third trimester is more than 3-fold lower than the rate among the ones receiving the first dose in the second trimester (0.3% vs.1%). Unless one assumes that the timing of vaccination greatly affects pregnancy outcomes, this is primarily the result of the fundamentally different gestational age at treatment.

## Intention to treat

The second bias in the analysis stems from the failure to control for the intention to treat. In a clinical trial, the treatment vs. placebo (control) assignment is determined at the time of recruiting, and if a negative outcome occurred before the treatment is administered, it will not be accounted towards the control. However, in the study of Dick et al., a woman is part of the control group until (if at all) she is vaccinated. Thus, if a negative outcome occurred prior to vaccination it is going to be assigned to the unvaccinated women. This creates another fundamental reverse correlation between the outcomes and the exposure, i.e., not having an early negative outcome, increases the likelihood to get vaccinated. Arguably there is a relatively small number of women who experienced a negative outcome prior to their intended day of vaccination. However, since the overall number of negative outcomes in the study is relatively small, this could still have meaningful impact on the results.

Considering the two biases discussed above, it is possible to assess the expected stillbirth rates in vaccinated and unvaccinated women, under the assumption that the vaccine has a neutral effect on the respective pregnancy outcomes. Given different assumptions on the temporal patterns of vaccination, the expected stillbirth rate among vaccinated women should be 2.5-4-fold lower than the respective rate among unvaccinated (see the excel based risk calculator in Supplementary material 1). Even under a relatively conservative assumption that 50% of all women who received the first dose in the second trimester received their second dose prior to week 22 (i.e., bear the full risk of a stillbirth), it is expected that the stillbirth rate among vaccinated women would be 2.75-fold lower than the unvaccinated. Thus, the fact that the observed rates among the vaccinated and unvaccinated women are similar is in fact somewhat concerning.

## Exclusion criteria

In addition to the biases discussed above, there is an additional bias in the analysis that stems from the exclusion criteria. In particular, in the exclusions of the study, Dick et al. mention that there are 22 women who were excluded from the analysis because they received their first dose prior to becoming pregnant.

Arguably, the exclusion of women who are vaccinated before pregnancy is a valid study design decision. However, under such a design decision, one has to exclude women based on the timing of pregnancy and not based on vaccination status. Note that the 22 vaccinated women that were excluded had by design a very short pregnancy below 28 weeks, since they became pregnant in January 2021 at the earliest and gave birth before end of July 2021. At the same time unvaccinated women who became pregnant in January 2021 and gave birth before end of July were not excluded. Indeed, by definition all of these births are pre-term (births prior to week 28), and there are all reasons to believe that many of them had negative outcomes including specifically stillbirth.

Recall, that according to the IMOH report [3] only 0.2% of live births and 51% of stillbirths occur prior to week 28. This implies that in all likelihood a large fraction of these 22 births were stillbirths. Note that among the 12 women in the study who received first dose in the first trimester (i.e., were at early stages of pregnancy in January), 6 had a stillbirth. Thus, even a conservative assumption that 11 out of the 22 that were excluded were stillbirths implies that the observed rate of stillbirths among the vaccinated women is higher than the unvaccinated. The unilateral exclusion of vaccinated women but not unvaccinated is highly problematic because these are women who by definition had the worse outcomes.

Another important potential issue is whether there are fundamental differences between the vaccinated and unvaccinated women that could affect the respective pregnancy outcomes. The reported baseline characteristics of vaccinated and unvaccinated women in Dick et al. seem quite similar, which means that they are not likely to explain the unexpected observed rates. Finally, it is interesting to note that the stillbirth and preterm rates in the population studied by Dick et al. are somewhat higher than the multi-year averages reported by IMOH [4] (5.9% vs. 4.6-5% and 0.94% vs. 0.6–0.75%, preterm and stillbirth rates, respectively).

To conclude, it is important to more explicitly acknowledge the potential biases in the retrospective study presented by Dick et al., since they could have a meaningful complementary impact on the interpretation of the results. This is particularly important to inform future studies that would hopefully better control for these biases. Moreover, the biases in the study by Dick et al. do exist in additional retrospective studies on the impact of COVID-19 vaccination during pregnancy on maternal and neonatal outcomes. In the absence of any data from clinical trials, these studies have served as the primary basis to inform public health recommendations. Finally, to have a more comprehensive interpretation of the results in Dick et al., it is important to report the pregnancy outcomes of the 22 vaccinated women that were excluded from the study because they received their first dose prior to conception, as well as the outcomes of women who received only one vaccine dose.

## Electronic supplementary material

Below is the link to the electronic supplementary material.

Supplementary Material 1

## Data Availability

Per request

## Footnotes

1 Based on personal communication.
